# Edible Plant-Derived Xanthones as Functional Food Components for Metabolic Syndrome Mitigation: Bioactivities and Mechanisms

**DOI:** 10.3390/foods14132344

**Published:** 2025-07-01

**Authors:** Dilireba Shataer, Shaohua Chen, Yaodan Wu, Fen Liu, Haipeng Liu, Jing Lu, Bailin Li, Liyun Zhao, Sheng-Xiang Qiu, Aikebaier Jumai

**Affiliations:** 1 College of Smart Agriculture (Research Institute), Xinjiang University, Urumqi 830046, China; dlrb_shataer2021@xju.edu.cn (D.S.); jinglu@xju.edu.cn (J.L.); 2 Key Laboratory of National Forestry and Grassland Administration on Plant Conservation and Utilization in Southern China, South China Botanical Garden, Chinese Academy of Sciences, Guangzhou 510650, China; chensh@scbg.ac.cn (S.C.); wuyaodan@scbg.ac.cn (Y.W.); sixpoints0518@163.com (F.L.); libailin@scbg.ac.cn (B.L.); zhaoly@scbg.ac.cn (L.Z.); sxqiu@scbg.ac.cn (S.-X.Q.); 3 University of Chinese Academy of Sciences, Beijing 100049, China; 4 Centre for Intelligent Healthcare, Coventry University, Coventry CV1 5RW, UK; haipeng.liu@coventry.ac.uk

**Keywords:** functional foods, metabolic syndrome, xanthones, bioactive compounds, food matrices, nutraceuticals

## Abstract

Metabolic syndrome has emerged as a significant global public health concern worldwide, characterized by a cluster of interrelated risk factors such as hypertension, hyperlipidemia, hyperglycemia, and abdominal obesity. In recent years, functional foods containing bioactive phytochemicals have attracted considerable scientific interest as potential therapeutic approaches for metabolic syndrome management. Xanthones, a class of naturally occurring tricyclic phenolic compounds abundant in various fruits and medicinal plants, demonstrate diverse biological activities relevant to metabolic health. This comprehensive review examines the dietary sources of xanthones, their bioactivity, and their promising role as functional food components for mitigating metabolic syndrome. The underlying mechanisms of action include modulation of lipid metabolism, improvement of insulin signaling pathways, potent anti-inflammatory and antioxidant effects, and modulation of glucose metabolism. Additionally, we discuss the stability and processing considerations of xanthones in food products. These findings highlight the development of xanthone-enriched functional foods and nutraceuticals as dietary interventions for metabolic syndrome prevention and management. This review comprehensively covers all relevant studies published up to the present without time restrictions.

## 1. Introduction

The definition of metabolic syndrome (MetS) has evolved over the years due to the challenges in establishing universally accepted diagnostic criteria. Nevertheless, MetS is broadly characterized by a cluster of metabolic abnormalities, including hypertension, central obesity, insulin resistance, and dyslipidemia [1,2,3]. The prevalence of MetS is rising globally, paralleling increases in obesity and type 2 diabetes (T2DM). In the United States, approximately one third of adults have MetS, with the prevalence of T2DM reaching 12.2% among adults, and even higher rates in seniors and certain ethnic groups. In China, urban MetS prevalence rose from 8% in 1992 to an estimated 15.5% in 2017. Globally, obesity rates have doubled in over 70 countries since 1980, contributing to the growing burden of MetS. While exact global MetS data remain limited, it is estimated that over one quarter of the world population—more than one billion people—are affected [4,5]. Effectively managing this multifaceted condition necessitates therapeutic strategies that simultaneously target its various components.

**Figure 1:**
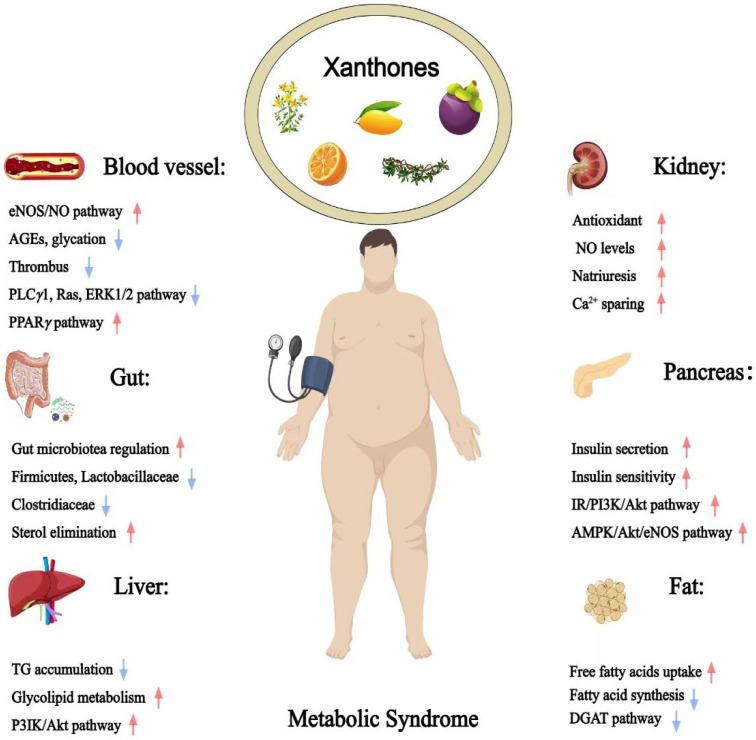
Therapeutic potential of xanthones against metabolic syndrome (Red arrows = activate; blue arrows = inhibit).

Xanthones, a class of polyphenolic compounds predominantly found in plants such as *Garcinia*, *Calophyllum*, *Hypericum*, *Platonia*, *Mangifera*, *Gentiana*, and *Swertia*, have attracted considerable scientific interest due to for their diverse pharmacological activities [6]. Their structural diversity and bioactive potential make them promising candidates for therapeutic intervention against metabolic syndrome and its associated pathologies [7,8,9]. Due to their presence in commonly consumed edible plants and demonstrated bioactivities, xanthones represent a promising category of functional food components that may contribute to the prevention and management of metabolic syndrome, thereby warranting a focused and comprehensive review [10]. However, previous reviews have predominantly focused on either the structural diversity or the biological activity of naturally occurring xanthones, often limiting their scope to specific genera like as *Garcinia* and *Mangifera*, or a select few well-studied compounds like *α*/*γ*-mangostin and mangiferin [6,11,12]. Given these limitations, a comprehensive overview examining edible plant-derived xanthones and their multifactorial roles in managing MetS is needed (Figure 1).

This review aims to provide a thorough analysis of the therapeutic potential and mechanistic insights of edible plant-derived xanthones in managing MetS. By exploring their multifactorial roles in alleviating the core components of MetS, we seek to elucidate the mechanisms underlying their beneficial effects. The review is systematically structured to address each component of metabolic syndrome individually, covering their impact on hypertension, lipid metabolism and obesity, and glucose homeostasis. First, we examine the role of xanthones in modulating hypertension, discussing evidence for their vasodilatory, antithrombotic, endothelial function-enhancing, and diuretic effects, which collectively contribute to blood pressure reduction and decreased cardiovascular risk. Next, we evaluate their impact on hyperlipidemia and obesity, emphasizing their ability to regulate lipid and energy metabolism, inhibit fatty acid synthesis, and prevent atherosclerosis. Subsequent sections explore the anti-hyperglycemic properties of xanthones, detailing their mechanisms in enhancing insulin secretion and sensitivity, delaying carbohydrate digestion and absorption, promoting glucose uptake, and increasing glucose excretion. By integrating mechanistic insights with pharmacological evidence, this review aims to advance the understanding of how edible plant-derived xanthones serve as effective therapeutic agents for metabolic syndrome.

## 2. Materials and Methods

A comprehensive and systematic literature search was conducted across multiple electronic databases, including Web of Science, PubMed, ScienceDirect, and Scopus, covering all publications up to April 2025. The search employed an advanced strategy combining the term in the topic “xanthone” with each of the following keywords using the Boolean operator AND: “metabolic syndrome”, “hypertension”, “hyperlipidemia”, “hyperglycemia”, “obesity”, “endothelial dysfunction”, “atherosclerosis”, “vasodilation”, “platelet aggregation”, “antithrombotic”, “dyslipidemia”, “hypoglycemic”, “antihyperglycemic”, “antidiabetic”, “insulin resistance”, “antihypertensive”, and “atherosclerosis”. This approach ensured retrieval of studies specifically examining the role of xanthones in metabolic and cardiovascular-related conditions. The initial search yield 1284 potentially relevant studies. Following the application of the predefined exclusion criteria, 176 studies eligible for inclusion in this review were left. Figures were prepared using InDraw and MedPeer platform (https://www.medpeer.cn; accessed on 16 May 2024).

### 2.1. Inclusion Criteria

(1) Studies published in peer-reviewed journals.
(2) Studies investigating the pharmacological properties and mechanisms of edible plant-derived naturally occurring xanthones in relation to metabolic syndrome.
(3) Studies published in the English language.

### 2.2. Exclusion Criteria

(1) Studies on synthetic xanthones which are not naturally occurring in plants.
(2) Studies on xanthone extracts without clarifying the components and structure.
(3) Studies published in a language other than English.
(4) Review articles, meta-analyses, case reports, and patents.

The search strategy included articles published up to April 2025. Additionally, the reference lists of relevant articles were carefully examined to identify any supplementary studies that might have been missed in the initial search. Strict inclusion and exclusion criteria were established to ensure only high-quality studies were included in this review.

## 3. Xanthones Against Hypertension

### 3.1. Vasodilatory Effects

**Figure 2:**
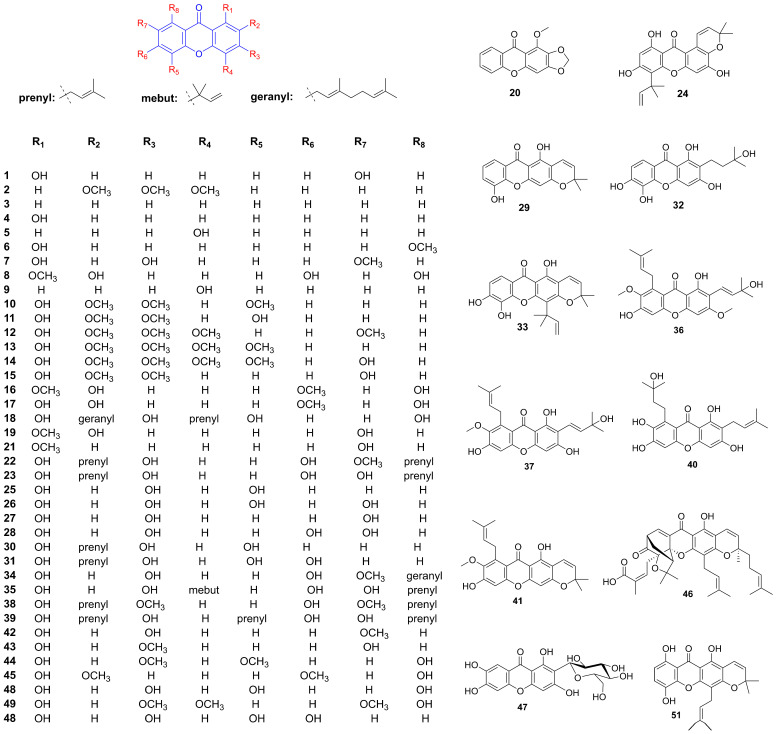
Structure of xanthones related to hypertension improvement.

The methanol extract of *Hypericum revolutum* Vahl, containing euxanthone (**1**) and 2,3,4-trimethoxy-xanthone (**2**) (Figure 2 and Table 1), demonstrated endothelium-dependent vasodilatory effects in isolated aortae. This activity was mediated by enhanced endothelial nitric oxide release, as evidenced by diminished efficacy upon endothelium removal or nitric oxide synthase inhibition with L-NAME [13,14]. Compounds 9-Xanthone (**3**), 1-hydroxyxanthone (**4**), 4-hydroxyxanthone (**5**), 1-hydroxy-8-methoxyxanthone (**6**), 1,3-dihydroxy-7-methoxyxanthone (**7**), 2,6,8-trihydroxy-1-methoxyxanthone (**8**), and 4-methoxyxanthone (**9**) demonstrated concentration-dependent vasorelaxant effects in endothelium-intact mouse aortic rings. Structural analysis suggested that a hydroxy group at the C-1 position attenuated vasodilation, whereas a hydroxy group at C-4 and an increased number of hydroxy groups enhanced xanthone-mediated vasorelaxation [15,16,17]. 1-Hydroxy-2,3,5-trimethoxyxanthone (**10**) isolated from *Halenia elliptica* induced relaxation in rat coronary artery rings via endothelium-dependent nitric oxide pathways and calcium channel inhibition. In contrast, 1,5-dihydroxy-2,3-dimethoxyxanthone (**11**) elicited endothelium-independent vasorelaxation through potassium channel modulation and partial inhibition of calcium influx [18,19]. Further investigations on six major xanthones 1-hydroxy-2,3,5-trimethoxy-xanthone (**10**), 1-hydroxy-2,3,4,7-tetramethoxyxanthone (**12**), 1-hydroxy-2,3,4,5-tetramethoxy-xanthone (**13**), 1,7-dihydroxy-2,3,4,5-tetramethoxyxanthone (**14**), 1,5-dihydroxy-2,3-dimethoxyxanthone (**11**), and 1,7-dihydroxy-2,3-dimethoxyxanthone (**15**) isolated from *Halenia elliptica* revealed vasodilatory activity in pre-contracted arteries through both endothelium-dependent and independent mechanisms [20]. Euxanthone (**1**) promoted vasodilation by inhibiting protein kinase C-activated calcium-sensitive mechanisms, independent of intracellular calcium release or voltage-operated calcium channels. It also induced concentration-dependent relaxation of high potassium and norepinephrine-induced contractions [21,22]. Gentiacaulein (**16**) and gentiakochianin (**17**), isolated from the methanolic extract of the roots of *Gentiana kochiana*, exhibited a vasorelaxant activity in rat aortic preparations pre-contracted with 3 µM norepinephrine [23]. 3-Demethyl-2-geranyl-4-prenylbellidifoline (**18**), a natural xanthone from *Garcinia achachairu*, reduced blood pressure in spontaneously hypertensive rats via endothelium-dependent vasorelaxation mediated by nitric oxide pathways and potassium channels [24]. Compounds 2,7-dihydroxy-1-methoxyxanthone (**19**), 1-methoxy-2,3-methylenedioxyxanthone (**20**), 7-hydroxy-1-methoxyxanthone (**21**), and euxanthone (**1**), isolated from the roots of *Polygala caudata*, displayed dose-dependent vasodilatory activity in Wistar rat thoracic aorta rings [25]. *α*-Mangostin (**22**) significantly improved endothelium-dependent vasodilation in diabetic mice by suppressing the acid sphingomyelinase/ceramide pathway and promoting the eNOS/NO pathway in the aorta [26]. Additionally, it reduced blood pressure and early nephropathy markers in spontaneously hypertensive rats, potentially by downregulating Ang II and inhibiting EMT via the TGF-*β* signaling pathway [27]. *γ*-Mangostin (**23**) induced concentration-dependent vasorelaxation in rat aortic rings precontracted with methoxamine, mediated by the NO-cGMP pathway and potassium channel activation [28].

### 3.2. Antiplatelet Aggregation Effects

Cudraxanthone B (**24**) effectively inhibited collagen-induced platelet aggregation, calcium ion mobilization, fibrinogen binding, fibronectin adhesion, and clot retraction without inducing cytotoxicity. It exhibited significant suppression of human platelet activation and thrombus formation [29]. 1,3,5-Trihydroxyxanthone (**25**) and 1,3,5,7-tetrahydroxyxanthone (**26**), isolated from *Garcinia cantleyana* var. cantleyana twigs, demonstrated selective inhibitory activity against ADP-induced platelet aggregation, with inhibition rates of 82.9 ± 8.8% and 90.4 ± 5.6%, respectively [30]. *α*-and *γ*-mangostin (**22**, **23**) demonstrated concentration-dependent inhibition of platelet aggregation induced by collagen, thrombin, and ADP at low concentrations (1–10 μM) [31]. *γ*-Mangostin (**23**) also functions as a competitive antagonist for 5-HT2A receptors in vascular smooth muscles and platelets. It selectively inhibited 5-HT-induced contractions in rabbit aorta and attenuated the perfusion pressure response of rat coronary artery to 5-HT. Additionally, *γ*-mangostin (**23**) inhibited ADP-amplified platelet aggregation [32]. Nine xanthones—4-hydroxyxanthone (**5**), 1,3,7-trihydroxyxanthone (**27**), 1,3,6,7-tetrahydroxyxanthone (**28**), 6-deoxyjacareubin (**29**), 2-(3-methylbut-2-enyl)-1-,3,5-trihydroxyxanthone (**30**), 2-(3-methylbut-2-enyl)-1,3,5,6-tetrahydroxyxanthone (**31**), 2-(3-hydroxy-3-methylbutyl)-1,3,5,6-tetrahydroxyxanthone (**32**), macluraxanthone (**33**), and rubraxanthone (**34**)—inhibited platelet aggregation induced by arachidonic acid, collagen, and adenosine diphosphate, with IC_50_ values ranging from 47.0 ± 3.5 μM to 247.8 ± 6.3 μM. The presence of a prenyl group at C-2 enhanced their antiplatelet activity, whereas cyclization or hydroxylation of the prenyl group increased the IC_50_ value [33]. Cudratricusxanthone A (**35**) exhibited antithrombotic activities by inhibiting fibrin polymerization, platelet aggregation, and the activities of thrombin and activated factor X. It significantly prolonged partial thromboplastin activation time, prothrombin time, and in vivo bleeding time [34]. Euxanthone (**1**), extracted from *Garcinia hombroniana* with methanol, exhibited significant and selective inhibition of ADP-induced platelet aggregation, with an IC_50_ value of 5.7 µm [35].

### 3.3. Endothelial Protective Effects

The deposition of advanced glycation end-products (AGEs) in tissues can lead to severe complications, including endothelial dysfunction, cardiovascular disorders, and atherosclerosis. Eight xanthones—mangostanaxanthones III (**36**) and IV (**37**), *β*-mangostin (**38**), garcinone E (**39**), rubraxanthone (**34**), *α*-mangostin (**22**), garcinone C (**40**), and 9-hydroxycalabaxanthone (**41**)—isolated from *Garcinia mangostana* L., demonstrated potent, dose-dependent inhibition of protein glycation induced by both sugar (ribose) and methylglyoxal [36]. Among these, *α*-mangostin (**22**) exhibited protective effects against diabetic vascular complications by reducing apoptosis in HUVECs and improving retinal microvascular health. It also displayed antihyperglycemic, antioxidant, anti-inflammatory, and antiglycation properties. Furthermore, it mitigates isoproterenol-induced myocardial necrosis through antioxidant mechanisms and modulation of the nitric oxide pathway [37,38,39,40]. Isogentisin (**42**), derived from *Gentiana lutea*, protected human vascular endothelial cells against death induced by cigarette smoke, H_2_O_2_, and UV exposure. It activated cellular repair functions, reduces protein oxidation, and stabilizes the microtubule system, contributing to cell shape maintenance [41]. Gentisin (**43**), also from *Gentiana lutea*, was identified as a novel inhibitor of vascular smooth muscle cell (VSMC) proliferation, with an IC_50_ value of 7.84 µM. Additionally, 1-hydroxy-2,3,4,5-tetramethoxyxanthone (**13**), swerchirin (**44**), and methylswertianin (**45**) exhibited moderate anti-proliferative activity against VSMC proliferation, with IC_50_ values ranging from 10.2 to 12.5 µM, and were confirmed to be non-toxic [42]. Gambogic acid (**46**) Inhibited proliferation, migration, and DNA synthesis in PDGF-BB-stimulated rat aortic VSMCs by inducing G_0_/G_1_ phase arrest and downregulating cell cycle regulators. It blocked PDGFR-*β* and related signaling pathways, and reversed TGF-*β*1-induced fibrotic transitions via the VASH-2/VASH-1 and TGF-*β*1/Smad3 pathways [43,44]. Mangiferin (**47**) enhanced endothelial function by enhancing exosome secretion from perivascular adipose tissue (PVAT) to attenuate inflammation via NF-*κ*B. It protected HUVECs against doxorubicin (DOX)-induced oxidative stress by upregulating Nrf2 through the PI3K/AKT pathway and ameliorates endoplasmic reticulum (ER) stress-related damage. Additionally, mangiferin exerts anti-atherosclerotic effects by reducing lipid accumulation and inflammatory responses in high-fat diet (HFD)-induced mice and improves endothelial cell responses to ox-LDL via the PTEN/Akt/eNOS pathway. Under hyperglycemic conditions, mangiferin activated Nrf2 and promoted angiogenesis and endothelial resilience, while in hyperuricemic rats, it alleviated hypertension and endothelial dysfunction [45,46,47,48,49,50]. Demethylbellidifolin (**48**), a xanthone derivative from *Swertia davidi* Franch, attenuated ox-LDL-induced monocyte adhesion to endothelial cells, reduced TNF-*α* and asymmetric dimethylarginine (ADMA) levels, and enhanced dimethylarginine dimethylaminohydrolase (DDAH) activity. It demonstrated antioxidant properties by inhibiting LDL oxidation and scavenging free radicals, while improving endothelium-dependent vasodilation and reducing lipid peroxidation [51,52]. Daviditin A (**49**) effectively preserved endothelium-dependent relaxation and mitigated lysophosphatidylcholine-induced endothelial dysfunction. It reduced lactate dehydrogenase release, malondialdehyde levels, and ADMA concentration, while increasing NO content and maintaining DDAH activity in ECV304 cells [53].

### 3.4. Diuretic Effects

1,3,5,6-Tetrahydroxyxanthone (**50**) exhibited diuretic activity along with a Ca^2+^-sparing effect, and reduced crystal formation in urine. It also modulated renal antioxidant and nitric oxide levels in hypertensive rats. Furthermore, 1,3,5,6-Tetrahydroxyxanthone showed synergistic enhancement of diuretic activity when co-administered with hydrochlorothiazide [54,55]. The diuretic effects of two natural prenylated xanthones, 3-demethyl-2-geranyl-4-prenylbellidypholine (DGP) (**18**) and 1,5,8-trihydroxy-4′,5′-dimethyl-2H-pyrano (2,3:3,2)-4-(3-methylbut-2-enyl) xanthone (TDP) (**51**), were evaluated in both normotensive and hypertensive rats. DGP exhibited potassium-sparing and diuretic properties, whereas TDP induced diuresis and increased urinary sodium, chloride, and calcium levels. When combined with hydrochlorothiazide, both DGP and TDP exhibited enhanced diuretic effects. Additionally, TDP showed protective effects against urinary calcium oxalate crystal formation [56]. DGP exhibits prolonged diuretic and nephroprotective effects in rats, promoting natriuresis, Ca^2+^ sparing, and antioxidant activity in spontaneously hypertensive rats [57].

## 4. Xanthones Against Hyperlipidemia and Obesity

### 4.1. Promoting Lipid Metabolism

**Figure 3:**
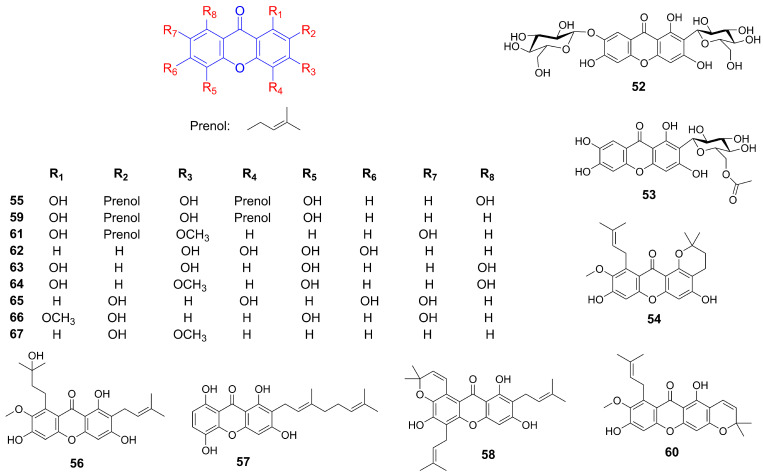
Structure of additional xanthones related to hyperlipidemia improvement.

Mangiferin (**47**) promoted cholesterol efflux and attenuates atherogenesis by activating the PPARγ-LXRα-ABCA1/G1 pathway, and improves fatty acid metabolism in HepG2 cells by enhancing *β*-hydroxybutyrate levels, free fatty acid (FFA) uptake, and oxidation via the AMPK pathway [58,59]. In non-alcoholic fatty liver disease (NAFLD) models, mangiferin (**47**) mitigated liver injury, insulin resistance, and glucose intolerance by modulating glucolipid metabolism through AMPK activation and NLRP3 inflammasome inhibition [60]. In HFD-induced mice, it reduced body weight, triglycerides, and total cholesterol while suppressing inflammation and enhancing autophagy via the AMPK/mTOR pathway [61]. In hyperlipidemic hamsters, mangiferin (**47**) reduced body and liver weight, visceral fat, serum triglycerides, and free fatty acids by upregulating lipid oxidation genes and downregulating lipogenesis genes [62]. When combined with exercise in KK-Ay mice, it lowered blood cholesterol and triglyceride levels, while in fructose-fed spontaneously hypertensive rats, it reduced hepatic triglyceride content by inhibiting diacylglycerol acyltransferase (DGAT-2) [63,64]. Furthermore, in human mesenchymal stem cells (hMSCs), mangiferin (**47**) suppressed adipocyte differentiation and lipid accumulation by downregulating key adipogenic genes [65]. Moreover, mangiferin (**47**) also exhibited antioxidant properties, suppressing SREBP-mediated lipogenesis, and inhibits AGE-induced oxidative stress. In HFD mice, it enhanced mitochondrial capacity, thermogenesis, glucose, and insulin profiles, shifting fuel utilization from fatty acids to carbohydrates [66]. In cultured myotubes, it increased glucose and pyruvate oxidation, ATP production, and targets pyruvate dehydrogenase [67]. Neomangiferin (**52**) effectively ameliorated HFD-induced NAFLD in rats by decreasing body weight, liver fat, serum lipids, and glucose levels while enhancing serum HDL cholesterol and hepatic antioxidant levels, These effects are mediated by the upregulation of PPAR*α* and CPT1a, and the downregulation of FATP2 and ACSL1 [68]. 6′-*O*-acetyl mangiferin (**53**) significantly reduced intracellular lipid and triglyceride accumulation in 3T3-L1 preadipocytes by activating AMPK [69]. In Apoe (−/−) mice, *α*-mangostin (**22**) attenuated atherosclerotic progression by reducing body weight gain, improving lipid profiles, and promoting M2 macrophage polarization. In Sprague Dawley rats, it alleviated HFD-induced hepatic steatosis by decreasing plasma-free fatty acids and liver triglycerides, improving antioxidant activity, and restoring mitochondrial function. Its potent antioxidant effects were demonstrated by prolonging the lag phase of LDL oxidation and reducing oxidative stress markers [70,71,72]. In HFD-induced obese mice, *α*-mangostin (**22**) improved metabolic profiles and obesity-related parameters by decreasing body, liver, and fat weights, as well as glucose, insulin, triglycerides, cholesterol, and fatty acid levels. Concurrently, it increased adiponectin, reducing inflammatory markers, improving glucose tolerance, and upregulating hepatic AMPK, SirT1, and PPARγ [73,74]. In diet-induced metabolic syndrome rats, it reduces body weight, abdominal fat, and improves liver and cardiovascular function by decreasing inflammation, collagen deposition, and reducing adipocyte size [75]. Additionally, *α*-mangostin (**22**) demonstrated anti-inflammatory and anti-adipogenic effects in 3T3-L1 preadipocytes by suppressing NF-*κ*B and adiponectin, while *γ*-mangostin (**23**) reduced adipogenesis and inflammation by inhibiting Nrf2 activity and PPARγ expression [76]. Several xanthones isolated from *Garcinia mangostana* (Figure 3), including *α*-mangostin (**22**), *β*-mangostin (**38**), *γ*-mangostin (**23**), 1-isomangostin (**54**), gartanin (**55**), garcinone D (**56**), 9-hydroxycalabaxanthone (**41**), smeathxanthone A (**57**), tovophyllin A (**58**), 8-deoxygartanin (**59**), mangostanin (**60**), and 1,7-dihydroxy-3-methoxy-2-(3-methylbut-2-enyl) xanthen-9-one (**61**) exhibited inhibitory activities against pancreatic lipase with IC_50_ ranging from 5.0 to 34.5 *µ*M (compared to orlistat IC_50_ 3.9 µM) [77]. 3,4,5,6-Tetrahydroxyxanthone (**62**) ameliorated dyslipidemia in ApoE (−/−) mice by lowering plasma and hepatic lipids, while increasing HDL cholesterol via modulation of Angptl3 and LPL. It also improved erythrocyte deformability and reduced oxidative stress markers, MDA, and ADMA, indicating potential benefits for lipid metabolism and vascular health [78,79]. A high-throughput screening identified 1,3,5,8-tetrahydroxyxanthone (**63**) as an agonist that enhances the interaction between ABCA1 and apoA-I, promoting cholesterol efflux in ABCA1-expressing cells and THP-1 macrophages, as confirmed by flow cytometry, Western blot, and 3H cholesterol efflux assays. This compound also dose-dependently inhibited lipid accumulation in 3T3-L1 adipocytes by reducing PPAR*γ* and C/EBP*α* expression while activating Hedgehog signaling components Gli1 and Smo [80,81]. Bellidifolin (**64**) significantly improves HFD-induced obesity and lipid disorders by reducing the abundance of *Firmicutes*, *Lactobacillaceae*, and *Clostridiaceae* in the gut microbiota, while enhancing bile acid biosynthesis and excretion to promote lipid metabolism [82].

### 4.2. Inhibiting Fatty Acid Synthesis

Mangiferin (**47**) supplementation (150 mg/day for 12 weeks) significantly improved serum lipid profiles in overweight hyperlipidemic patients by reducing serum TG and FFA, while enhancing lipoprotein lipase activity and markers of fatty acid oxidation including L-carnitine and *β*-hydroxybutyrate. The reduction in lowering serum TG levels may result from either the inhibition of TG synthesis or the acceleration of TG decomposition [83]. Quantitative proteomic analysis revealed that mangiferin (**47**) significantly modulated 87 out of 865 liver proteins in mice fed a high-fat diet, specifically enhancing proteins involved in mitochondrial biogenesis and oxidative metabolism while downregulating those associated with lipogenesis. These findings suggested that mangiferin promotes energy expenditure while suppressing fat synthesis [84]. Garcinone E (**39**), a bioactive compound derived from *Garcinia mangostana*, demonstrated concentration-dependent inhibition of fatty acid synthase (FAS) with an IC_50_ of 3.3 μM. Mechanistic studies demonstrated competitive inhibition relative to acetyl-CoA, mixed competitive and noncompetitive inhibition with malonyl-CoA, and noncompetitive inhibition regarding NADPH. Notably, its irreversible inhibition of FAS highlight garcinone E’s potential as a therapeutic agent for obesity and cancer, where FAS plays a crucial role [85]. Natural inhibitors of FAS are emerging as promising candidates for cancer and obesity treatment. A bioassay-guided chemical investigation of *Garcinia mangostana* hulls led to the isolation of several xanthones, including *α*-mangostin (**22**), *β*-mangostin (**38**), *γ*-mangostin (**23**), 9-hydroxycalabaxanthone (**41**), garcinone E (**39**), 1,3,7-trihydroxyxanthone (**27**), and 2,4,6,7-tetrahydroxyxanthone (**65**). These compounds exhibited potent FAS inhibitory activity, with IC_50_ values ranging from 1.24 ± 0.05 to 40.98 ± 0.64 μM [86]. Additionally, the ethanolic extract of *Garcinia mangostana* peel inhibited human recombinant DGAT1, DGAT2, and pancreatic lipase (PL) activities in vitro. Among the tested bioactive compounds, *α*-mangostin (**22**), *γ*-mangostin (**23**), gartanin (**55**), and 8-deoxygartanin (**59**) were identified as potent inhibitors of DGAT enzymes [87]. Furthermore, *α*-mangostin (**22**) induced apoptosis in 3T3-L1 cells, suppressed FAS activity, inhibited lipid accumulation, and promoted lipolysis by targeting the ketoacyl synthase domain of FAS [88].

### 4.3. Anti-Atherosclerosis Effect

1-Methoxy-2,5,7-trihydroxyxanthone (**66**) isolated from the roots of *Lindera fruticose* demonstrated LDL antioxidant activity with an IC_50_ value of 25.5 µM (positive control, probucol, IC_50_ = 4.3 µM). Given that LDL cholesterol oxidation is a critical step in atherosclerotic lesion formation, this compound shows potential therapeutic relevance [89]. Cudratricusxanthone A (**35**), derived from the root bark of *Cudrania tricuspidata*, demonstrated antiproliferative effects on PDGF-BB-stimulated vascular smooth muscle cells. Its mechanism involves suppression of DNA synthesis, cell proliferation, PDGFR*β* phosphorylation, and downstream signaling pathways including PLCγ1, Ras, and ERK1/2 [90]. 3-Methoxy-2-hydroxyxanthone (**67**), isolated from *Calophyllum flavoramulum* Hend. & Wyatt-Sm, exhibited potent anti-AGEs activity with an IC_50_ value of 0.06 mM, suggesting its utility in mitigating advanced glycation end product-related pathologies [91]. Mangiferin (**47**) alleviated TMAO-induced atherosclerosis in mice by attenuating inflammation, reducing plasma cholesterol levels, and modulating gut microbiota composition. These effects collectively diminish aortic lesions and enhance the elimination of sterols [92].

### 4.4. Promoting Energy Metabolism

**Figure 4:**
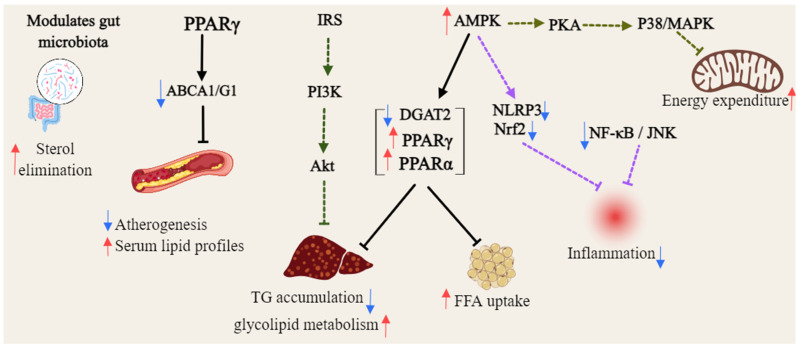
General overview of the mechanism of xanthones against hyperlipidemia (Red arrows = activate; blue arrows = inhibit; dashed lines = modulation).

Gambogic acid (**46**) activated AMPK by binding to the AMPK *α* subunit, independent of upstream kinases, and enhancing phosphorylation of AMPK *α* and its substrate ACC across multiple cell lines. This activation was uniquely mediated by intracellular ROS, with levels modulated by thiol antioxidants, suggesting its potential as a novel direct AMPK activator for metabolic diseases management [93]. Mangiferin (**47**) enhances brown fat markers, induces UCP1, and upregulates thermogenic regulators (PGC1*α*, PRDM16, PPAR-*α*/*β*). It promotes mitochondrial biogenesis and function by activating AMPK and *β*3-AR-dependent PKA-p38 MAPK-CREB signaling, while suppressing mitophagy [95,96]. *α*-Mangostin (**22**) mitigated oxidative stress and metabolic disorders by enhancing AMPK phosphorylation [94]. *γ*-mangostin (**23**) was identified as an active compound that induced UCP-3 gene expression in L6 cells, functioning as a dual agonist for PPAR*δ* and PPAR*α*. Additionally, it upregulated acyl-CoA synthase and carnitine palmitoyl-transferase 1A gene expression in HepG2 cells, indicating its potential as a preventive agent for metabolic syndrome [97], as shown in Figure 4.

## 5. Xanthones Against Hyperglycemia

### 5.1. Increasing Insulin Secretion

**Figure 5:**
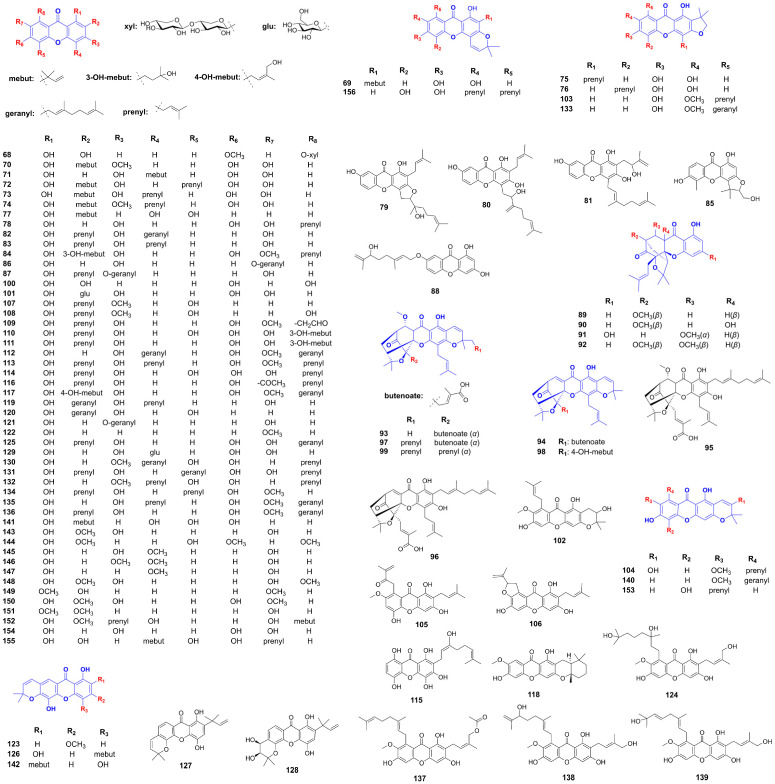
Structure of additional xanthones related to hyperglycemia improvement.

Supplementation with *α*-mangostin (**22**) in diabetic rats significantly reduced fasting blood glucose, HbA1c, cholesterol, and triglyceride levels, while enhancing insulin secretion and sensitivity, as well as improving glucose tolerance. Mechanistically, *α*-mangostin stimulated insulin release in INS-1 cells by activating the IR, Pdx1, PI3K, Akt, and ERK pathways, while suppressing IRS-1 phosphorylation [98,99]. In a 28-day study, gentiakochianin (**17**) and 1,2-dihydroxy-6-methoxyxanthone-8-*O*-*β*-D-xylopyranosyl (**68**) isolated from *Swertia corymbosa* demonstrated significant antidiabetic and antihyperlipidemic effects, markedly reducing blood glucose, glycosylated hemoglobin, and lipid profiles (Figure 5). These compounds also improved liver and kidney function markers while elevating plasma insulin levels [100]. Mangiferin (**47**) demonstrated significant improvements in glycemic control, glucose tolerance, serum, and insulin levels in diabetic models. It also promoted *β*-cell hyperplasia, proliferation, and reduced apoptosis. Furthermore, treatment with a hydroalcoholic *Bombax ceiba* leaf extract and its constituent mangiferin (**47**) improved glucose regulation, lipid metabolism, and oxidative stress markers in streptozotocin-induced diabetic rats, likely through increased insulin release, antioxidant activity, and hypolipidemic effects [101,102]. A 30-day mangiferin (**47**) treatment (40 mg/kg) in diabetic rats ameliorated blood glucose levels, enhanced insulin secretion, and improved nonenzymatic antioxidant capacity (vitamins C, E, ceruloplasmin, reduced glutathione). Histological analysis revealed regenerated islet cells and normalized pancreatic ultrastructure, confirming *β*-cell protection [103]. The combination of sitagliptin with mangiferin (**47**) synergistically improved glucose tolerance, insulin, and GLP-1 levels in diabetic rats, highlighting its potential for metabolic control. In aged, partially pancreatectomized mice, mangiferin reduced blood glucose, enhanced glucose tolerance, increased insulin production, and promoted *β*-cell proliferation, while suppressing apoptosis via the modulation of cyclins and cyclin-dependent kinases [104,105]. Crude swerchirin (**44**) from *Swertia chirayita* induced a 60% reduction in blood glucose levels in fed rats by potentiating insulin release from pancreatic islets, as evidenced by both in vivo and in vitro studies. Notably, swerchirin treatment depleted *β*-granules and insulin in pancreatic islets while simultaneously promoting glucose uptake and glycogen synthesis in muscle cells [106].

### 5.2. Enhancing Insulin Sensitivity

In Wistar-Kyoto rats fed a fructose diet, mangiferin (**47**) reduced plasma insulin and non-esterified fatty acid (NEFA) levels, mitigating adipose tissue insulin resistance without affecting fructose intake, fat accumulation, or hypertension [107]. In HFD mice, it alleviated endoplasmic reticulum (ER) stress, suppressed NLRP3 inflammasome activation, enhanced AMPK activity, and restored insulin-mediated Akt and eNOS phosphorylation, thereby improving endothelial function and presenting a potential therapeutic approach for preventing endothelial insulin resistance [108]. For gestational diabetes, mangiferin (**47**) improved glucose and lipid metabolism, reduced placental oxidative stress, increased antioxidant levels, and attenuated inflammation and ER stress [109]. In KK-Ay mice, administration of mangiferin (47) at a 30 mg/kg dose decreased serum insulin levels, enhanced glucose uptake, and promoted fatty acid oxidation [110]. In palmitic acid-treated cells, mangiferin (**47**) enhanced insulin-stimulated glucose uptake while reducing intracellular glucose, FFA, and TG levels in by upregulating p-Akt, GLUT2, and GLUT4 expression, and by activating fatty acid oxidation via the PPAR pathway [111]. In hypoxic 3T3-L1 adipocytes, it counteracts insulin resistance by inhibiting HIF-1*α* [112]. In diabetic nephropathy models, mangiferin (**47**) provided renoprotective effects by inhibiting the MAPK/NF-*κ*B axis, activating the phosphorylated IRS-1/PI3K/Akt pathway, and mitigating renal ferroptosis through enhanced antioxidant activity and reduced pro-ferroptotic lipid production [113]. In type 2 diabetes models, mangiferin (**47**) improved biochemical parameters, modulated lipid metabolism, and ameliorated obesity, hyperglycemia, insulin resistance, and dyslipidemia. It restored hepatic glycogen levels, normalized serum TNF-*α* and adiponectin concentrations, and demonstrated efficacy comparable to rosiglitazone [114,115]. When combined with metformin, mangiferin (**47**) targeted the insulin-dependent Akt pathway, whereas its co-administration with gliclazide affected the insulin-independent AMPK pathway [116]. Additionally, mangiferin (**47**) and neomangiferin (**52**) significantly reduced blood glucose levels and improved hyperinsulinemia in non-insulin-dependent diabetes mellitus models, highlighting their potential to enhance insulin sensitivity [117]. *α*-Mangostin (**22**) and *γ*-mangostin (**23**), derived from mangosteen, reduced LPS-induced inflammation and counteracted aging-related metabolic disorders in mice by reducing pro-inflammatory cytokines, promoting macrophage polarization toward anti-inflammatory phenotypes, and suppressing microRNA-155-5p secretion. These compounds exhibit anti-inflammatory effects in human adipocytes and macrophages by inhibiting MAPK, NF-*κ*B, and AP-1 pathways, thereby reducing LPS-induced inflammatory gene expression and enhancing insulin sensitivity [118,119,120]. Chemical investigation of *Cudrania tricuspidata* roots yielded nine xanthones, including cudratricusxanthone N (**69**), 1,6,7-trihydroxy-2-(1,1-dimethyl-2-propenyl)-3-methoxyxanthone (**70**), cudratricusxanthone L (**71**), cudratricusxanthone A (**35**), cudraxanthone L (**72**), macluraxanthone B (**73**), cudracuspixanthone A (**74**), cudraxanthone D (**75**), and cudraxanthone M (**76**). These prenylated xanthones exhibited dose-dependent PTP1B inhibitory activity with IC_50_ values ranging from 1.9 to 4.6 μM [121]. 12b-Hydroxy-des-D-garcigerin A (**77**) improved glucose consumption in insulin-resistant HepG2 cells by increasing hexokinase and pyruvate kinase activities while upregulating key insulin signaling components, including InsR, IRS-1, phosphorylated PI3K, and phosphorylated AKT, without affecting AMPK phosphorylation at Thr172 [125]. 1,3,6,7-Tetrahydroxy-8-prenylxanthone (**78**) isolated from *Garcinia mangostana* attenuated LPS-induced inflammation in macrophages and adipocytes by activating MAPKs and NF-*κ*B pathways and enhancing sirtuin 3 expression [126]. Methylswertianin (**45**) and bellidifolin (**64**) significantly improved hyperglycemia, lipid profiles, and insulin signaling in streptozotocin-induced type 2 diabetic mice by upregulating InsR-*α*, IRS-1, and PI3K expression [127]. Bellidifolin (**64**), obtained from *Swertia japonica*, exhibited potent hypoglycemic effects and reduced blood triglyceride levels in diabetic rats, while stimulating glucose uptake in Rat 1 fibroblasts expressing human insulin receptors [128]. Twelve xanthones—including cratoxanthone E (**79**), cratoxanthone F (**80**), cratoxanthone A (**81**), cochinechinone A (**82**), 1,3,7-trihydroxy-2,4-diisoprenylxanthone (**83**), *α*-mangostin (**22**), *γ*-mangostin (**23**), cratoxylone (**84**), cochinchinone Q (**85**), 7-geranyloxy-1,3-dihydroxyxanthone (**86**), pruniflorone S (**87**), and cochinxanthone A (**88**)—isolated from *Cratoxylum cochinchinense* demonstrated dual inhibition of PTP1B (IC_50_ 2.4–52.5 µM) and *α*-glucosidase (IC_50_ 1.7–72.7 µM) [122]. Four new caged xanthones—cochinchinoxanthone A (**89**), B (**90**), C (**91**), and D (**92**) —from *Cratoxylum cochinchinense* roots acted as competitive PTP1B inhibitors [123]. Additionally, the methanol extract of *Garcinia hanburyi* gum resin, containing prenylated caged xanthones, such as gambogic acid (**46**), moreollic acid (**93**), morellic acid (**94**), 10-methoxygambogenic acid (**95**), gambogenic acid (**96**), gambogoic acid (**97**), morellinol (**98**), and 10-methoxygambogin (**99**), exhibited dose-dependent PTP1B inhibition (IC_50_ 0.47–70.25 μM), with gambogic acid, demonstrating superior potency to the positive control, ursolic acid (IC_50_ 15.5 μM) [124].

### 5.3. Slowing Digestion and Absorption

Several xanthones derived from *Swertia mussotii*, including 1,3,7,8-tetrahydroxyxanthone (**100**), 1,3,5,8-tetrahydroxyxanthone (**63**), and 2,3,6,8-tetrahydroxyxanthone-7C-(*β*-D-glucoside) (**101**), exhibited notable antioxidant activity and *α*-glucosidase inhibition. Specifically, 1,3,5,8-tetrahydroxyxanthone (**63**) displayed potent dual inhibition of *α*-glucosidase (IC_50_ = 5.2 ± 0.3 μM) and aldose reductase (IC_50_ = 88.6 ± 1.6 nM) [129]. *α*-Mangostin (**22**) exhibited strong antioxidant properties alongside inhibitory effects on *α*-amylase and *α*-glucosidase. Mechanistic studies indicated that *α*-mangostin (**22**) reversibly interacts with these enzymes, particularly through hydrogen bonding with *α*-glucosidase [142]. Similarly, *γ*-mangostin (**23**), a xanthone derived from *Garcinia mangostana*, demonstrated significant antihyperglycemic effects in diet-induced diabetic mice, effectively lowering blood glucose levels while exhibiting superior inhibitory activity against *α*-amylase and *α*-glucosidase compared to acarbose, without hepatotoxic or nephrotoxic effects [143]. The ethanol extract of *Garcinia mangostana* fruit case, rich in xanthones, showed potent *α*-glucosidase inhibition (IC_50_ = 3.2 μg/mL). Isolated xanthones—including *β*-mangostin (**38**), 9-hydroxycalabaxanthone (**41**), mangostanol (**102**), mangostenone F (**103**), allanxanthone E (**104**), *α*-mangostin (**22**), mangostingone (**105**), garcinone D (**56**), *γ*-mangostin (**23**), mangosenone G (**106**), cudraxanthone (**107**), 1,5,8-trihydroxy-3-methoxy-2-(3-methylbut-2-enyl)xanthone (**108**), 8-deoxygartanin (**59**), gartanin (**55**), smeathxanthone A (**57**), and oxoethylmangostine (**109**)—demonstrated moderate-to-high *α*-glucosidase inhibition, with IC_50_ values ranging from 1.5 to 63.5 μM [130]. Further studies identified five xanthones—mangostanaxanthone VIIII [1,3,5,6,7-pentahydroxy-2-(3-methylbut-2-enyl)-8-(3-hydroxy-3-methylbut-1-enyl)-xanthone] (**110**), mangostanaxanthone I (**111**), mangostanaxanthone II (**112**), *γ*-mangostin (**23**), and mangostanaxanthone VII (**113**)—from the acetone fraction of *Garcinia mangostana* pericarps, showing *α*-amylase inhibition ranging from 56.2% to 86.55% [146]. Additionally, 8-deoxygartanin (**59**), *α*-mangostin (**22**), and *β*-mangostin (**23**) exhibited polyfunctional inhibition against pancreatic lipase, *α*-amylase, and *α*-glucosidase, with IC_50_ values ranging from 31.6 ± 2.6 to 333.5 ± 4.5 μM [144]. A newly identified xanthone, garcixanthone D [1,3,5,6,7-pentahydroxy-2,8-bis(3-methylbut-2-enyl)-xanthone] (**114**), along with garcinone E (**39**) from *Garcinia mangostana*, exhibited the highest *α*-amylase inhibition at 85.6% and 93.8%, respectively [147,148]. Another novel xanthone, garcimangostin A (**115**), along with known compounds garcixanthone A (**116**), gartanin (**55**), *γ*-Mangostin (**23**), and garcinone C (**40**), also inhibited *α*-amylase, with inhibition percentages ranging from 54.9% to 94.1% [149]. Screening of *Garcinia cowa* leaves revealed that *α*-mangostin (**22**) and cowanol (**117**) displayed significant *α*-glucosidase inhibition, with IC_50_ values of 15.0 μM and 18.0 μM, respectively [131]. Six xanthones—garciniacowone L (**118**), 2-geranyl-1,3,7-trihydroxy-4-(3,3-dimethylallyl)-xanthone (**119**), mangostinone (**120**), cochinchinone G (**121**), 1-hydroxy-7-methoxyxanthone (**122**), and forbexanthone (**123**)—isolated from *Garcinia cowa* Roxb. ex Choisy exhibited *α*-glucosidase inhibition (IC_50_ = 85.1 ± 0.3 to 188.8 ± 0.6 μM), comparable to acarbose (IC_50_ = 76.7 ± 1.4 μM) [132]. Similarly, garcicowanones C (**124**), cowanol (**117**), norcowanin (**125**), and garcinone D (**56**) from *Garcinia cowa* exhibited *α*-glucosidase inhibition (IC_50_ = 17.2 ± 0.3 to 211.2 ± 3.4 μM, acarbose, 257.3 ± 4.8 μM) [133]. Four xanthones—subelliptenone H (**126**), 12b-hydroxy-des-D-garcigerin A (**76**), garciniaxanthone B (**127**), and garcigerin A (**128**)—isolated from *Garcinia forbesii*, inhibited *α*-glucosidase and *α*-amylase with an IC_50_ value ranging from 10.8 ± 0.04 to 139.4 ± 1.21 μM (Acarbose, 4.0 ± 0.32 μM) [145]. The water extract of *Anemarrhena asphodeloides* rhizoma (AA) significantly reduced blood glucose levels in KK-Ay mice, primarily due to mangiferin (**47**) and neomangiferin (**52**). Mangiferin (**47**) exhibited strong *α*-glucosidase inhibition (IC_50_ = 36.84 μg/mL) in both in silico and in vitro studies [134,135]. Furthermore, when combined with metformin and gliclazide, mangiferin (**47**) significantly alleviated renal injury in type II diabetic rats by reducing serum glucose, urea, and creatinine levels, while modulating inflammation and oxidative stress-related markers [136]. Mangiferin (**47**) also inhibited DPPH radicals and α-glucosidase [137,138]. Xanthone-enriched fractions from *Cyclopia genistoides*, along with mangiferin (**47**) and isomangiferin (**129**), showed potent *α*-glucosidase inhibition (IC_50_ = 43.3 µg/mL) and synergistic effects when combined with acarbose [139]. From *Garcinia oblongifolia*, oblongixanthones C (**130**), F (**131**), G (**132**), and H (**133**), 1,3,6-trihydroxy-7-methoxy-2,5-bis(3-methylbut-2-enyl)xanthon (**134**), isocowanin (**135**), cowanin (**136**), cowanol (**117**), rubraxanthone (**34**), cowagarcinone E (**137**), and norcowanin (**125**) exhibited significant *α*-glucosidase (IC_50_ = 1.7 to 53.5 μM) and PTP1B inhibition (IC_50_ = 14.1 to 44.2 μM) [151]. Two additional xanthones, oblongixanthones I (**138**) and J (**139**), showed moderate *α*-glucosidase (IC_50_ = 258.7 ± 49.3 μM and 187.1 ± 27.5 μM) and PTP1B inhibition (IC_50_ = 93.9 ± 12.3 μM and 64.1 ± 5.8 μM) [152]. Among 18 xanthones isolated from *Garcinia nigrolineata* Planch. ex, cowagarcinone E (**137**) exhibited the strongest *α*-glucosidase inhibitory activity (IC_50_ = 25.8 ± 0.2 µM), while mangostanin (**60**) and fuscaxanthone A (**140**) showed the highest *α*-amylase inhibitory activity (IC_50_ = 124.8 ± 0.7 µM) and glycation inhibition activity (IC_50_ = 44.4 ± 1.1 µM), respectively [150]. From *Garcinia xanthochymus* bark, subelliptenone F (**141**) and xanthochymusxanthone B (**142**) significantly inhibited *α*-glucosidase (IC_50_ = 4.1 ± 0.3 μM) and PTP1B (IC_50_ = 8.0 ± 0.6 μM) [153]. *Securidaca inappendiculata* Hassk., traditionally used for fractures and rheumatoid arthritis, contains xanthones with antidiabetic properties. Eleven xanthones—1,3,7-trihydroxylxanthone (**27**), 1,3,7-trihydroxyl-2-methoxylxanthone (**143**), 1,5-dihydroxyl-2,6,8-trimethoxylxanthone (**144**), 1,3,7-trihydroxyl-4-methoxylxanthone (**145**), 1,7-dihydroxyl-3,4-dimethoxylxanthone (**146**), 1,7-dihydroxyl-4-methoxylxanthone (**147**), euxanthone (**1**), 1,3,7-trihydroxyl-2,8-dimethoxylxanthone (**148**), 2-hydroxyl-1,7-dimethoxylxanthone (**149**), 1,3,6-trihydroxyl-2,7-dimethoxylxanthone (**150**), and 7-hydroxyl-1,2-dimethoxylxanthone (**151**)—inhibited *α*-glucosidase (IC_50_ = 3.2 to 77.3 μg/mL), with free hydroxyl groups playing a key role in activity [140]. Garceduxanthone (**152**) and 7-prenyljacareubin (**153**) isolated from the leaves of *Garcinia paucinervis* showed moderate *a*-glucosidase inhibition (IC_50_ = 8.90 ± 3.35 and 29.36 ± 0.81 μM, respectively), compared to acarbose (IC_50_ = 2.88 ± 0.85 μM) [141].

### 5.4. Enhancing Glucose Uptake

Mangiferin (**47**), isolated from the leaves of *Salacia oblonga*, was administered to streptozotocin-induced diabetic rats and significantly reduced blood glucose levels, normalized lipid profiles, and improved oxidative stress biomarkers over a 15-day period. Molecular docking and gene expression analyses confirmed that mangiferin activates the PPAR*γ*/GLUT4 signaling pathway, suggesting its therapeutic potential in enhancing glucose uptake and ameliorating diabetes symptoms [154]. Additionally, *Salacia oblonga* extract, particularly its active component mangiferin (**47**), enhanced glucose uptake by 50% in muscle L6-myotubes and 3T3-adipocytes. This effect was mediated through increased GLUT4 content, activation of GLUT4 transcription, and facilitation of its translocation via the activation of AMPK. The process was modulated through PPAR*γ*, as evidenced by inhibition with the antagonist GW9662, indicating dual independent pathways for glucose transport enhancement [155]. In streptozotocin-induced diabetic rats, mangiferin (**47**) also demonstrated significant antidiabetic, antihyperlipidemic, and antiatherogenic effects by reducing fasting plasma glucose, total cholesterol, triglycerides, and LDL-C levels while increasing HDL-C levels and improving glucose tolerance [156]. Targeted metabolomics and transcriptomics studies demonstrated that mangiferin (**47**) enhanced glycolytic flux and mitochondrial oxidative capacity. These studies reported increases in glycolytic metabolites and key tricarboxylic acid cycle intermediates, such as *α*-ketoglutarate and fumarate. Mangiferin also upregulated succinate dehydrogenase, improving ATP production, and induced the expression of mitochondrial genes and their transcription factors, suggesting its role in accelerating metabolic processes [157]. Furthermore, mangiferin (**47**) from *Salacia chinensis*, administered at a dose of 40 mg/kg body weight daily for 30 days, significantly reduced blood glucose and improved biochemical markers including urea, uric acid, and creatinine in streptozotocin-induced diabetic rats. It also decreased toxicological parameters such as AST, ALT, and ALP while enhancing hematological profiles [158]. Norathyriol (**154**) and mangiferin (**47**) significantly enhanced glucose consumption in L6 myotubes, with norathyriol (**154**) exhibiting a more pronounced effect, particularly in insulin-resistant cells. Both compounds acted synergistically with insulin and their antidiabetic activity was mediated through the upregulation of AMPK phosphorylation [159]. *α*-Mangostin (**22**) inhibited adipogenic differentiation, reduced intracellular fat accumulation, enhanced glucose uptake, and altered gene expression related to PPAR*γ*, GLUT4, and leptin [160]. *Garcinia xanthochymus* is a folk medicine widely used in southwestern China. Compounds 12b-hydroxy-des-d-garcigerrin (**76**), 1,2,5,6-tretrahydroxy-4-(1,1-dimethyl-2-propenyl)-7-(3-methyl-2-butenyl) xanthone (**155**), and 1,5,6-trihydroxy-7,8-di(3-methyl-2-butenyl)-6′,6′-dimethylpyrano (2′,3′:3,4) xanthone (**156**) were isolated from *G. xanthochymus* and significantly stimulate glucose uptake in skeletal muscle cells. The first two compounds induced GLUT4 translocation through activation of PI3K/Akt and AMPK pathways [161].

### 5.5. Effects Against Inflammation and Oxidative Stress

**Figure 6:**
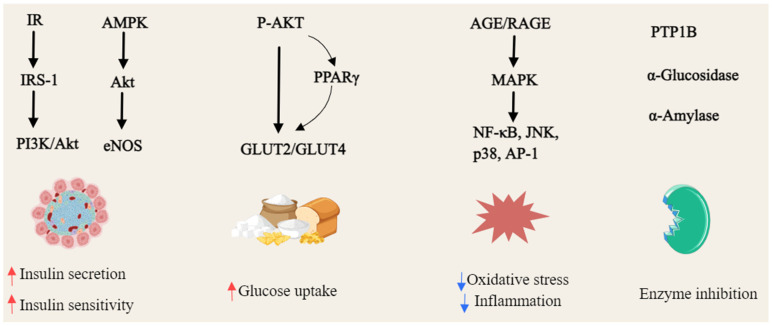
General overview of the mechanism of xanthones against hyperglycemia (Red arrows = activate; blue arrows = inhibit).

*α*-Mangostin (**22**) reversed high glucose-induced inhibition of proliferation and migration in HUVECs. RNA-seq analysis revealed that it restored the expression of H19 and HE4 lncRNAs, which were down-regulated under hyperglycemic conditions. Further studies demonstrated that H19 modulates HE4 expression via miR-140, suggesting a novel H19/miR-140/HE4 regulatory pathway for *α*-mangostin’s potential therapeutic impact in diabetes mellitus [162]. *γ*-Mangostin (**23**) treatment significantly reduced fasting blood glucose, cholesterol, SGOT, and SGPT levels, while mitigating hepatocyte damage in STZ-induced diabetic BALB/C mice [176]. Mangiferin (**47**), first isolated from the root bark of *Salacia reticulata* in 1985, exhibited multiple therapeutic effects in diabetic models. In diabetic rats, it ameliorated cardiomyopathy by reducing myocardial enzyme levels (CK-MB, LDH), inflammatory mediators (TNF-*α*, IL-1*β*), and reactive oxygen species (ROS), while suppressing the AGE/RAGE pathway and NF-*κ*B nuclear translocation [163,164]. In ischemia–reperfusion (IR) injury models, mangiferin enhanced cardiac function, restored antioxidant defenses, and reduced inflammation and apoptosis by modulating the AGE-RAGE/MAPK pathways—specifically inhibiting JNK and p38 activation while upregulating ERK1/2 [165]. Additionally, it also improved renal function in diabetic nephropathy by enhancing glyoxalase 1 (Glo-1) activity, reducing albuminuria, and alleviating oxidative stress [166]. In STZ-induced diabetic rats, mangiferin (**47**) restored the suppressed hypoxic ventilatory response and normalized oxidative and inflammatory stress markers [167]. Network pharmacology analysis identified 37 key targets, including TNF, EGF, and PTGS2, implicating mangiferin (**47**) in critical pathways such as PI3K-Akt and NF-*κ*B [168]. Furthermore, it alleviated cognitive deficits in diabetic rats by increasing Glo-1 activity and glutathione levels while reducing oxidative stress and inflammation in the hippocampus [169]. Mangiferin (**47**) also attenuated diabetic nephropathy by decreasing serum AGEs and malondialdehyde, promoting antioxidant enzyme activity and inhibiting glomerular extracellular matrix expansion [170]. In STZ-induced diabetic Wistar rats, it provided significant cardiorenal protection against oxidative damage, reducing glycosylated hemoglobin and CPK levels comparably to insulin treatment [171]. Moreover, mangiferin (**47**) mitigated nephrotoxicity by inhibiting PKC isoforms, MAPKs, and NF-*κ*B activation [172]. In H9C2 cells, it protected against H_2_O_2_-induced ischemia–reperfusion injury by enhancing antioxidant capacity and metabolic pathways, revealing key enzyme targets and mechanistic insights into its cardioprotective effects [173]. The hypoglycemic activity of the alcoholic extract of *Swertia japonica* is primarily attributed to its xanthones, with bellidifolin (**78**) and swerchirin (**44**) significantly reducing blood glucose in STZ-induced diabetic rats [174]. Swerchirin (**44**), isolated from *Swertia chirayita*, exhibited potent glucose-lowering effects across various rat models, with an ED_50_ of 23.1 mg/kg for a 40% reduction in blood glucose [175], as shown in Figure 6.

## 6. Conclusions and Perspectives

In summary, edible plant-derived naturally occurring xanthones exhibit promising therapeutic potential in addressing various aspects of metabolic syndrome, including hypertension, hyperlipidemia, obesity, and hyperglycemia. Their pharmacological effects are mediated through diverse and interconnected molecular pathways including eNOS/NO/cGMP, AMPK/DGAT, PPAR*γ*, AMPK/PKA/p38 MAPK, AMPK/Akt/eNOS, and insulin receptor-related signaling (IR/IRS-1/PI3K/Akt). These mechanisms underlie observed biological activities including vasodilation, anti-platelet aggregation, endothelial protection, modulation of lipid metabolism, enhancement of energy expenditure, improved insulin secretion and sensitivity, and facilitation of glucose uptake and metabolism. The antioxidative and anti-inflammatory properties of xanthones may also contribute to mitigating oxidative stress and inflammation associated with metabolic dysregulation. Structural characteristics, including hydroxylation patterns and prenylation, significantly influence their efficacy in regulating vascular tone, inhibiting key enzymes (such as fatty acid synthase, protein tyrosine phosphatase 1B, and carbohydrate-digesting enzymes), and activating cellular protective mechanisms. Among the xanthones reviewed, compounds such as *α*-mangostin and mangiferin have been most extensively studied, demonstrating consistent effects in both in vitro and in vivo models, as well as limited clinical investigations. These studies suggest potential benefits on endothelial function, lipid accumulation, insulin resistance, and diabetic complications.

Recent advances in delivery systems, including nanotechnology-based approaches, offer possible strategies to improve the bioavailability and pharmacokinetic profiles of xanthones, which could enhance their therapeutic utility. However, further research is needed to clarify their safety, efficacy, and mechanisms at molecular and systemic levels. Integrative approaches such as multi-omics and network pharmacology may help dissect the complex interactions underlying their activity. Moreover, the integration of emerging artificial intelligence (AI) and machine learning techniques offers a powerful opportunity to advance the identification of novel molecular targets, predict compound–target interactions, and elucidate complex mechanisms of action. AI-driven computational modeling and virtual screening can accelerate structure–activity relationship analyses and facilitate the design of optimized xanthone derivatives with improved efficacy and selectivity. Combining AI approaches with experimental validation presents a promising strategy to overcome traditional bottlenecks in drug discovery and to enhance the precision of translational research on bioactive natural products. Additionally, optimizing structure–activity relationships and formulation technologies could support the development of more potent and selective derivatives. Critically, well-designed clinical trials are essential to substantiate the therapeutic potential of xanthones in metabolic syndrome management, including determination of effective dosing, safety profiles, and possible drug interactions. Examining potential synergistic effects with established treatments and lifestyle interventions might also provide more comprehensive management strategies.

In conclusion, substantial evidence underscores the potential of naturally derived xanthones as versatile agents capable of modulating key pathophysiological pathways in metabolic syndrome. Their incorporation into future therapeutic strategies holds promise for advancing the clinical management of this multifaceted condition. However, realizing this potential requires sustained multidisciplinary research to bridge experimental findings with clinical translation, ultimately enabling the development of safe, effective, and accessible xanthone-based interventions for metabolic health improvement.

## Figures and Tables

**Table 1:** Pharmacological activities of xanthones and its mechanism of action.

Mechanism Category	Key Mechanisms	Xanthones	References
Anti-hypertension			
Vasodilation	NO-dependent vasodilation	Euxanthone, 2,3,4-trimethoxyxanthone, 1-hydroxy-2,3,5-trimethoxyxanthone, 3-demethyl-2-geranyl-4-prenylbellidifoline, *α*-mangostin, *γ*-mangostin	[13,14,18,24,26,28]
	Concentration-dependent vasorelaxant	9-Xanthenone, 1-hydroxyxanthone, 4-hydroxyxanthone, 1-hydroxy-8-methoxyxanthone, 1,3-dihydroxy-7-methoxyxanthone, 2,6,8-trihydroxy-1-methoxyxanthone, 4-methoxyxanthone, 1-hydroxy-2,3,4,7-tetramethoxyxanthone, 1-hydroxy-2,3,4,5-tetramethoxyxanthone, 1,7-dihydroxy–2,3,4,5-tetramethoxyxanthone, 1,7-dihydroxy-2,3-dimethoxyxanthone, gentiacaulein, gentiakochianin, 2,7-dihydroxy-1-methoxyxanthone, 1-methoxy-2,3-methylenedioxyxanthone, 7-hydroxy-1-methoxyxanthone	[15,16,17,20]
	Ca^2+^ channel inhibition	1-Hydroxy-2,3,5-trimethoxyxanthone	[18]
	K^+^ channel modulation	1,5-Dihydroxy-2,3-dimethoxyxanthone, 3-demethyl-2-geranyl-4-prenylbellidifoline, *γ*-mangostin	[19,28]
Anti-platelet aggregation	Inhibitory activity against collagen, thrombin, and ADP	Cudraxanthone B, 1,3,5-trihydroxyxanthone, 1,3,5,7-tetrahydroxyxanthone, *α/γ*-mangostin, 4-hydroxyxanthone, 1,3,7-trihydroxyxanthone, 1,3,6,7-tetrahydroxyxanthone, 6-deoxyjacareubin, 2-(3-Methylbut-2-enyl)-1-,3,5-trihydroxyxanthone, 2-(3-Methylbut-2-enyl)-1,3,5,6-tetrahydroxyxanthone, 2-(3-hydroxy-3-methylbutyl)-1,3,5,6-tetrahydroxyxanthone, macluraxanthone, rubraxanthone, cudratricusxanthone A, euxanthone	[29,30,31,32,33,34,35]
Endothelial protection	Inhibit protein glycation	mangostanaxanthones III, mangostanaxanthones IV, *β*-mangostin, garcinone E, rubraxanthone, *α*-mangostin, garcinone C, 9-hydroxycalabaxanthone	[36,37,38,39,40]
	Activates cellular repair functions	Isogentisin	[41]
	Inhibit VSMC proliferation	Gentisin, 1-hydroxy-2,3,4,5-tetramethoxyxanthone, swerchirin, methylswertianin, gambogic acid	[42,43,44]
	PVAT exosomes mitigate stress via NF-*κ*B, Nrf2	Mangiferin	[45,46,47,48,49,50]
	Reduces monocyte adhesion, oxidative stress, and improves vasodilation	Demethylbellidifolin	[51,52]
	Preserves relaxation, reduces oxidative damage, boosts NO	Daviditin A	[53]
Diuretic effects	Enhances diuresis, spares Ca^2+^, reduces crystals	1,3,5,6-Tetrahydroxyxanthone	[54,55]
	Induces diuresis, increases sodium, chloride, calcium, protective against calcium oxalate crystals	3-Demethyl-2-geranyl-4-prenylbellidypholine	[56,57]
	Potassium-sparing, diuretic, prolongs kidney protection, promotes natriuresis, Ca^2+^ sparing, antioxidant	1,5,8-Trihydroxy-4′,5′-dimethyl-2H-pyrano (2,3:3,2)-4-(3-methylbut-2-enyl) xanthone	[56]
Anti-hyperlipidemia and obesity			
Promoting lipid metabolism	Activating the PPARγ-LXRα-ABCA1/G1 pathway Activating AMPK pathway	Mangiferin	[58,59,60,61,62,63,64,65,66,67]
	Upregulation of PPAR*α* and CPT1a, and downregulation of FATP2 and ACSL1	Neomangiferin	[68]
	Activating AMPK	6′-*O*-acetyl mangiferin	[69]
	Activates AMPK, Sirt1, and PPARγ, reducing inflammation and enhancing lipid catabolism.	*α*-Mangostin	[70,71,72,73,74]
	Inhibitory activities against pancreatic lipase	*α/β/γ*-mangostin, 1-isomangostin, gartanin, garcinone D, 9-hydroxycalabaxanthone, smeathxanthone A, tovophyllin A, 8-deoxygartanin, mangostanin, 1,7-dihydroxy-3-methoxy-2-(3-methylbut-2-enyl) xanthen-9-one	[75,76,77]
	Modulates Angptl3 and LPL	3,4,5,6-Tetrahydroxyxanthone	[78,79]
	Activates ABCA1, reduce PPAR*γ* and C/EBP*α* expression	1,3,5,8-Tetrahydroxyxanthone	[80,81]
	Gut microbiota modulation, enhances bile acid synthesis and excretion	Bellidifolin	[82]
Inhibiting fatty acid synthesis	Activates mitochondrial biogenesis and oxidative pathways	Mangiferin	[83,84]
	Competing with acetyl-CoA and malonyl-CoA	Garcinone E	[85]
	Inhibit fatty acid synthase enzyme	*α/β/γ*-Mangostin, 9-hydroxycalabaxanthone, 1,3,7-trihydroxyxanthone, 2,4,6,7-tetrahydroxyxanthone, gartanin, 8-deoxygartanin	[86,87,88]
Anti-atherosclerosis effect	Reducing oxidative modification of LDL cholesterol	1-Methoxy-2,5,7-trihydroxyxanthone	[89]
	Blocking DNA synthesis, PDGFRβ activation, and downstream PLCγ1, Ras, and ERK1/2 signaling pathways	Cudratricusxanthone A	[90]
	Anti-AGEs activity	3-Methoxy-2-hydroxyxanthone	[91]
	Modulating gut microbiota	Mangiferin	[92]
Promoting energy metabolism	Activates AMPK	Gambogic acid, *α*-mangostin	[93,94]
	Activates PKA-p38 MAPK-CREB signaling	Mangiferin (47)	[95,96]
	Activates PPAR*δ* and PPAR*α*	*γ*-Mangostin	[97]
Anti-hyperglycemia			
Increasing insulin secretion	Activating IR, Pdx1, PI3K, Akt, ERK pathways, inhibiting IRS-1 phosphorylation	*α*-Mangostin	[98,99]
	Restore insulin secretion, improve pancreatic function	Gentiakochianin, 1,2-dihydroxy-6-methoxyxanthone-8-*O*-*β*-D-xylopyranosyl	[100]
	Protecting pancreatic *β*-cells, promoting their proliferation, reducing apoptosis	Mangiferin	[101,102,103,104,105]
	Enhanced insulin release from pancreatic islets	Swerchirin	[106]
Enhancing insulin sensitivity	Activation of the Akt, AMPK signaling pathway Suppression of ER stress and NLRP3 inflammasome activation Inhibition of HIF-1*α* Modulation of the MAPK/NF-κB axis	Mangiferin	[107,108,109,110,111,112,113,114,115,116,117]
	Inhibiting the MAPK, NF-*κ*B, and AP-1 signaling pathways	*α/γ*-Mangostin	[118,119,120]
	PTP1B inhibition	Cudratricusxanthone N, 1,6,7-trihydroxy-2-(1,1-dimethyl-2-propenyl)-3-methoxyxanthone, cudratricusxanthone L, cudratricusxanthone A, cudraxanthone L, macluraxanthone B, cudracuspixanthone A, cudraxanthone D, cudraxanthone M cratoxanthone E, cratoxanthone F, cratoxanthone A, *α/γ*-mangostin, cratoxylone, cochinchinone Q, 7-geranyloxy-1,3-dihydroxyxanthone, pruniflorone S, cochinxanthone A, cochinchinoxanthone A/B/C/D, gambogic acid, moreollic acid, morellic acid, 10-methoxygambogenic acid, gambogenic acid, gambogoic acid, morellinol, 10-methoxygambogin	[121,122,123,124]
	UpregulatingInsR, IRS-1, p-PI3K, and p-AKT, increasing the activities of hexokinase and pyruvate kinase	12b-Hydroxy-des-D-garcigerin A	[125]
	Activating MAPKs and NF-κB pathways	1,3,6,7-Tetrahydroxy-8-prenylxanthone	[126]
	Upregulating InsR-*α*, IRS-1, and PI3K expression	Methylswertianin, bellidifolin	[127,128]
Slowing digestion and absorption	*α*-glucosidase inhibition	1,3,7,8-Tetrahydroxyxanthone, 1,3,5,8-tetrahydroxyxanthone, 2,3,6,8-tetrahydroxyxanthone-7C-(*β*-D-glucoside), 9-hydroxycalabaxanthone, mangostanol, mangostenone F, allanxanthone E, mangostingone, garcinone D, mangosenone G, cudraxanthone, 1,5,8-trihydroxy-3-methoxy-2-(3-methylbut-2-enyl)xanthone, 8-deoxygartanin, gartanin, smeathxanthone A, oxoethylmangostine, cowanol, garciniacowone L, 2-geranyl-1,3,7-trihydroxy-4-(3,3-dimethylallyl)-xanthone, mangostinone, cochinchinone G, 1-hydroxy-7-methoxyxanthone, forbexanthone, garcicowanones C, cowanol, norcowanin, mangiferin, neomangiferin, isomangiferin, cowagarcinone E, 1,3,7-trihydroxylxanthone, 1,3,7-trihydroxyl-2-methoxylxanthone, 1,5-dihydroxyl-2,6,8-trimethoxylxanthone, 1,3,7-trihydroxyl-4-methoxylxanthone, 1,7-dihydroxyl-3,4-dimethoxylxanthone, 1,7-dihydroxyl-4-methoxylxanthone, euxanthone, 1,3,7-trihydroxyl-2,8-dimethoxylxanthone, 2-hydroxyl-1,7-dimethoxylxanthone, 1,3,6-trihydroxyl-2,7-dimethoxylxanthone, 7-hydroxyl-1,2-dimethoxylxanthone, Garceduxanthone, 7-prenyljacareubin	[129,130,131,132,133,134,135,136,137,138,139,140,141]
	Inhibition of *α*-amylase and *α*-glucosidase enzymes	*α/β/γ*-Mangostin, 8-deoxygartanin, subelliptenone H, 12b-hydroxy-des-D-garcigerin A, garciniaxanthone B, garcigerin A	[142,143,144,145]
	*α*-amylase inhibition	Mangostanaxanthone VIIII, mangostanaxanthone I, mangostanaxanthone II, mangostanaxanthone VII, garcixanthone D, garcinone C/E, garcimangostin A, garcixanthone A, gartanin, mangostanin, fuscaxanthone A	[146,147,148,149,150]
	*α*-glucosidase and PTP1B inhibition	Oblongixanthone C/F/G/H, 1,3,6-trihydroxy-7-methoxy-2,5-bis(3-methylbut-2-enyl) xanthon, isocowanin, cowanin, cowanol, rubraxanthone, cowagarcinone E, norcowanin, oblongixanthones I/J, subelliptenone F, xanthochymusxanthone B	[151,152,153]
Enhancing glucose uptake	Activates PPAR*γ*/GLUT4 and AMPK pathways	Mangiferin	[154,155,156,157,158]
	Upregulating AMPK phosphorylation	Norathyriol	[159]
	Modulates expression of PPAR*γ*, GLUT4, and leptin genes	*α*-Mangostin	[160]
	Activating of PI3K/Akt and AMPK pathways	12b-Hydroxy-des-d-garcigerrin, 1,2,5,6-tretrahydroxy-4-(1,1-dimethyl-2-propenyl)-7-(3-methyl-2-butenyl) xanthone, 1,5,6-trihydroxy-7,8-di(3-methyl-2-butenyl)-6′,6′-dimethylpyrano (2′,3′:3,4) xanthone	[161]
Anti-inflammation and oxidative stress related to hyperglycemia	Modulate H19/miR-140/HE4 pathway	*α*-Mangostin	[162]
	Inhibited AGE/RAGE pathway and NF-*κ*B nuclear translocation; modulates the AGE-RAGE/MAPK pathways	Mangiferin	[163,164,165,166,167,168,169,170,171,172,173]
Others	dose depended hypoglycemic activity	Bellidifolin, swerchirin	[174,175]

## Data Availability

The data supporting this review are derived from previously reported studies and data sets, which have been cited. The processed data are available from the first author upon request.

## Abbreviations

The following abbreviations are used in this manuscript:

ADMA	Asymmetric dimethylarginine
ADP	Adenosine diphosphate
AGEs	Advanced glycation end-products
Ang II	Angiotensin II
cGMP	Cyclic guanosine monophosphate
DDAH	Dimethylarginine dimethylaminohydrolase
DGAT	Diacylglycerol acyltransferase
DGAT-2	Diacylglycerol acyltransferase
EMT	Epithelial-to-mesenchymal transition
eNOS	Endothelial Nitric Oxide Synthase
ER	Endoplasmic reticulum
FAS	Fatty acid synthase
FFA	Free fatty acids
GLUT4	Glucose transporter 4
HFD	High fat diet
hMSCs	Human mesenchymal stem cells
HUVEC	Human umbilical vein endothelial cells
LPC	Lysophosphatidylcholine
MetS	Metabolic syndrome
NAFLD	Non-alcoholic fatty liver disease
NEFA	Non-esterified fatty acid
NO	Nitric oxide
Nrf2	Nuclear factor erythroid 2-related factor 2
ox-LDL	Oxidized low-density lipoprotein
PDGF	Platelet-derived growth factor
PL	Pancreatic lipase
PTEN	Phosphatase and tension homologue
PTP1B	Human Protein Tyrosine Phosphatase 1B
ROS	Reactive oxygen species
TG	Triglycerides
TGF-β	Transforming growth factor-β
VSMC	Vascular smooth muscle cell
VASH	Vasohibin
5-HT	5-Hydroxytryptamine
